# Spleen Removal

**Published:** 1896-01

**Authors:** Jas. F. Duncan

**Affiliations:** Navasota, Texas


For the Texas Medical Journal.

### SPLEEN REMOVAL.

BY JAS. F. DUNCAN, M. D., NAVASOTA, TEXAS.

DR. J. A. BELL, assisted by Dr. Wm. Goodrich, both of Navasota, Texas, on the 21st day of November, 1895, removed the spleen of Katie Libscomb, colored; age, 21 years; married, and mother of two cnildren. She has lived the last five years in the Brazos river bottom, a very malarious district. She has had intermittent fever, at intervals, for some years About eighteen months ago she began to experience uneasiness in her left side, and observed that there was a hard enlargement there; this continued to increase until her physician, Dr. Bell, was consulted about it. He found the spleen much enlarged and displaced downward. He proposed an operation, to which the patient readily agreed. Having chloroformed his patient, the doctor made an incision along the center of the abdomen to the left of the umbilicus, about six inches in length. Finding this insufficient to allow the passage of the enlarged spleen, he cut at right angles to the first incision, six inches. He then was able to make a satisfactory exploration. He found the spleen with a number of abnormal attachments to the surrounding vicera, which he broke up, and, with a great caution, proceeded to remove the entire spleen; secondly, ligating all wounded blood vessels.

Having removed the organ, which weighed five pounds, and carefully sponged away all blood, he closed the wound in the abdomen with surgeon's silk. The patient rallied readily and nicely from the chloroform, and did quite well. I saw her on the twentieth day after the operation, and found her sitting up. She said she had no pain, and was able to sit up all day. The operation was performed in a little dirty cabin in the country. The wound healed by first intention. There was never any suppuration. The operation was under the modern antiseptic method. The only dressing was a fresh application of bichloride gauze with iodoform, absorbent cotton and bandage, changed every day for a week.

This is the first time this operation has been performed in this section of country, and as far as I know, in Texas. Dr. Bell has been in Texas only two years; is a young man from Mississippi, and a graduate of the Hospital College of Louisville, Ky., and Jefferson College, Philadeiphia.

